# Clinical and Surgical Outcomes in Extensive Scalp Reconstruction after Oncologic Resection: A Comparison of Anterolateral Thigh, Latissimus Dorsi and Omental Free Flaps

**DOI:** 10.3390/jcm10173863

**Published:** 2021-08-27

**Authors:** José Luis del Castillo Pardo de Vera, Carlos Navarro Cuéllar, Ignacio Navarro Cuéllar, José Luis Cebrián Carretero, Sandra Bacián Martínez, María Isabel García-Hidalgo Alonso, Arturo Sánchez-Pérez, Jose J. Zamorano-León, Antonio J. López-Farré, Carlos Navarro Vila

**Affiliations:** 1 Maxillofacial Surgery Department, Hospital La Paz, Paseo de la Castellana, 261, 28046 Madrid, Spain; delcastillo6@hotmail.com (J.L.d.C.P.d.V.); rodrigator2001@hotmail.com (J.L.C.C.); 2 Maxillofacial Surgery Department, General Universitario HLA Moncloa, Avenida de Valladolid, 83, 28008 Madrid, Spain; nnavcu@hotmail.com (I.N.C.); sbacian@gmail.com (S.B.M.); hazamachado2@gmail.com (C.N.V.); 3 Radiology Department, Hospital Puerta de Hierro, 28046 Madrid, Spain; mabelgha@gmail.com; 4 Faculty of Medicine, Murcia University, 30100 Murcia, Spain; arturosa@um.es; 5 Faculty of Medicine, Universidad Complutense de Madrid, 28046 Madrid, Spain; jjzamorano@ucm.es (J.J.Z.-L.); ajlf@telefonica.net (A.J.L.-F.)

**Keywords:** scalp reconstruction, anterolateral thigh flap, omental flap, latissimus dorsi flap

## Abstract

Microsurgical scalp reconstruction is indicated in patients with large scalp defects. The aim of this study was to compare the outcomes of scalp reconstruction in oncologic patients reconstructed with latissimus dorsi (LD), anterolateral thigh (ALT), and omental (OM) free flaps. Thirty oncologic patients underwent scalp reconstruction with LD (10), ALT (11), and OM (9) flaps. The length of the vascular pedicle, the operation time, the possibility of a two-team approach, the length of hospital stays, the complications, and the aesthetic results were evaluated. The OM flap was the flap with the shortest vascular pedicle length with a mean of 6.26 ± 0.16 cm, compared to the LD flap, which was 12.34 ± 0.55 cm and the ALT flap with 13.20 ± 0.26 cm (*p* < 0.05). The average time of surgery was 6.6 ± 0.14 h in patients reconstructed with OM, compared to the LD flap, which was 8.91 ± 0.32 h and the ALT flap with 7.53 ± 0.22 h (*p* < 0.05). A two-team approach was performed in all patients for OM flaps and ALT flaps, but only in two patients reconstructed with the LD flap (*p* < 0.001). In patients reconstructed with the OM flap, a very satisfactory or satisfactory result was reported in seven patients (77.8%). Eight patients reported a very unsatisfactory or unsatisfactory result with LD flap (80%) and 10 patients with ALT flap (90.9%) (*p* = 0.002). The mean hospital stay after surgery was not statistically significant (*p* > 0.05). As for complications, two patients reconstructed with OM flap, five LT flaps, and two ALT flaps developed complications, not statistically significant (*p* = 0.235). Omental flap, latissimus dorsi flap, and anterolateral thigh flap fulfill most of the characteristics for complex scalp reconstruction. The decision on which flap to use should be based on clinical aspects of the patients taking into account that the three flaps show similar rates of complications and length of hospital stay. Regarding the aesthetic outcome, OM flap or LD flap should be considered for reconstruction of extensive scalp defects.

## 1. Introduction

Scalp reconstruction after oncologic resection and radiation therapy remains a challenge in head and neck reconstruction owing to the specific vascularization patterns of the scalp and the poor elasticity of the soft tissues [1]. Primary closure, second intention healing, tissue expanders, skin grafts, and local and regional flaps are the classic methods for the reconstruction of defects that are not very extensive [2]. Because of the low donor site morbidity and the possibility to replace like to like with good aesthetic results, local flaps should be considered as the main reconstructive technique for scalp defects [3]. Nevertheless, patients with larger and full-thickness defects cannot be reconstructed by means of these techniques and microvascular flaps are needed [4]. Maxwell performed the first free latissimus dorsi flap (LD) for head and neck reconstruction supplied by the thoracodorsal artery and the advantages include the reliability of the surgical technique, the versatility and a long vascular pedicle, low morbidity, and primary closure [5]. The anterolateral thigh flap (ALT) is supplied by the descending branch of the lateral femoral circumflex artery, it enables to work in two operative fields and provides excellent soft tissue volume and a long and reliable vascular pedicle. Omental flap (OM) for scalp reconstruction was first reported by McLean and Buncke [6]. It is usually supplied by the right gastroepiploic artery, and it is harvested with a minimal invasive procedure laparoscopically. It provides large and pliable soft tissue for extensive scalp reconstruction, and it is covered by a split-thickness skin graft with good aesthetic result [7]. When reconstructing an extensive scalp defect in which local or regional flaps are not sufficient to reconstruct this defect, the question would be: which flap do we use to reconstruct these defects to achieve a good clinical and surgical result for our patients?

The aim of this retrospective study was to evaluate and compare the outcomes of scalp reconstruction in oncologic patients reconstructed with omental flap, latissimus dorsi, and anterolateral thigh free flaps in order to determine which flap has the major advantages for the reconstruction of extensive scalp defects. The investigators hypothesize that the omental flap has similar clinical results to the ALT and LD flaps and, although not a commonly used flap for scalp reconstruction, it should also be considered as a first-choice flap as well as the LD flap and the ALT flap for the reconstruction of extensive scalp defects. The specific aims of the study were to compare variables of interest between the three reconstructive techniques, such as the length of the vascular pedicle, the operation time, the possibility of a two-team approach, the complications, the length of hospital stay, and the aesthetic results.

## 2. Materials and Methods

### 2.1. Patient Selection

To address the research purpose, the investigators implemented a retrospective study with 30 oncologic patients who underwent scalp reconstruction with LD, ALT, and OM flaps between March 2010 and April 2019 at Hospital La Paz and HLA Moncloa General Hospital (Madrid, Spain). Informed consents were obtained from all patients. The study and review of the medical records and data collection, and the subsequent analysis of the data collected is endorsed by the Hospital Ethics Committee. The inclusion criteria were: (1) oncologic patients with extensive tumors affecting the scalp; (2) patients who underwent prior radiotherapy with scalp defects and bone exposure; (3) patients with recurrent tumors in the scalp that required further surgery at this level. The exclusion criteria were: (1) patients reconstructed with local or regional flaps; (2) patients with cranial defects or titanium mesh exposure that required patient-specific implants and/or free flaps.

Preoperative CT data were processed, and all patients were treated with wide resection of the tumor and reconstructed with either LD, ALT, or OM flaps. In patients who had previously undergone abdominal surgery, the omental flap was not considered as a reconstructive technique. In these cases, the LD flap or ALT flap were used for reconstruction. In patients who had previously undergone surgery on the axilla, LD flap was not considered for reconstruction. OM flap was harvested in patients with extensive scalp defects in which no prior abdominal surgery was performed.

Patient data were extracted from the medical records concerning age, sex, disease, flap size, team feasibility, type of flap, location defect, recipient vessels, and radiotherapy. The variables evaluated and compared between the three reconstructive techniques were: (a) the length of the vascular pedicle; (b) the operative time; (c) the possibility of a two-team approach; (d) the aesthetic result; (e) the complications; and (f) the length of hospital stay. The aesthetic result was evaluated at least one year after surgery and the results were classified with scores 1 (“very unsatisfactory”), 2 (“unsatisfactory”), 3 (“neutral”), 4 (“satisfactory”), or 5 (“very satisfactory”).

### 2.2. Statistical Analysis

Quantitative values were expressed as mean ± standard error of mean (S.E.M), while qualitative variables were expressed as frequencies and percentages. Kruskal–Wallis and Mann–Whitney’s tests were used to compare differences between groups of quantitative variables. Qualitative variables were compared using Chi-squared test. The statistical analysis was performed using the software SPSS 25.0. A two-tail *p* value < 0.05 was considered statistically significant.

## 3. Results

Thirty oncologic patients underwent scalp reconstruction with OM, LD, and ALT flaps. Twenty patients were male (66.6%) and 10 patients were female (33.3%), with a mean age of 63.63 ± 1.38 years. Location of the defects are described in Supplementary Tables S1–S3. Microvascular anastomoses were performed to the superficial temporal vessels in 19 patients (63.3%) and to the facial vessels in 11 patients (36.7%). There were no complications in 21 (70%) of the patients. Two patients reconstructed with OM flap developed partial skin graft failure treated conservatively. In the LD group, one patient developed a seroma in the donor site and a wound dehiscence was reported in two patients, while revision of the microvascular anastomosis was performed in one patient. In the ALT group, one patient developed a seroma and in one patient, a venous thrombosis was diagnosed 24 h after surgery and required an interpositional vein graft to perform an end-to-end anastomosis. Twelve patients received radiotherapy (40%), of which two were preoperative radiotherapy of previous tumors and the rest postoperative radiotherapy. A two-team approach was performed in 22 cases (73.3%). The mean pedicle length was 10.83 ± 0.60 cm, with a mean operation time of 7.71 ± 0.22 h and the mean hospital stay was 16.17 ± 0.70 days. The aesthetic result was reported as very satisfactory in one patient (3.3%), satisfactory in six patients (20%), neutral in five cases (16.7%), unsatisfactory in eight patients (26.7%), and very unsatisfactory in 10 patients (33.3%) (Table 1 and Table 2).

### 3.1. Omental Flap

Nine patients with scalp defects were reconstructed with the OM flap. Six patients were male (66.6%), and three patients were female (33.3%), with a mean age of 63 years (range 55 to 71 years). Pathology included squamous cell carcinoma (n = 6), basal cell carcinoma (n = 1), adenocarcinoma (n = 1), and melanoma (n = 1). The size of flaps ranged from 21 to 12 cm in width and 19 to 10 cm in length. The recipient vessels were the superficial temporal artery and vein (n = 8) and the facial vessels (n = 1). The survival rate of the flap was 100%. Two patients underwent radiotherapy prior to surgery (60 Gy). Two patients with partial skin graft necrosis were encountered and required further debridement and skin graft. A two-team approach was performed in all patients. The mean length of vascular pedicle was 6.26 ± 0.16 cm (range 5.8 to 7.3 cm). The mean operation time was 6.6 ± 0.14 h (range 6.1 to 7.5 h). The mean hospital stay was 15.78 ± 0.93 days (range 13 to 21 days) and the aesthetic result was reported as very satisfactory in one patient (11.1%), satisfactory in six patients (66.7%), and a neutral result in two patients (22.2%) (Table 3 and Table 4).

#### Case Presentation

**Figure 1:**
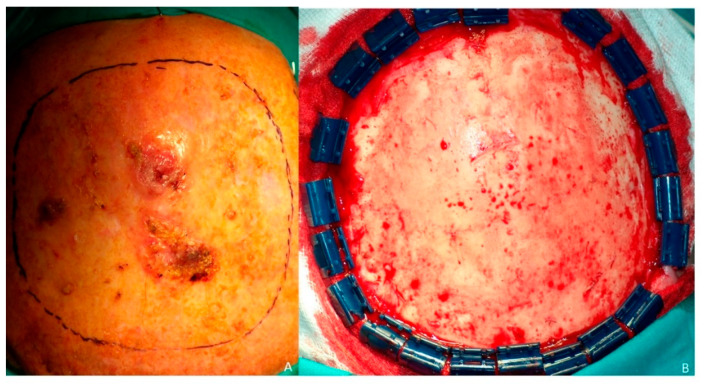
Omental flap. (**A**) Recurrent squamous cell carcinoma of the scalp. (**B**) Full-thickness defect of scalp after resection of the tumor and resection of the surrounding fibrous tissue.

**Figure 2:**
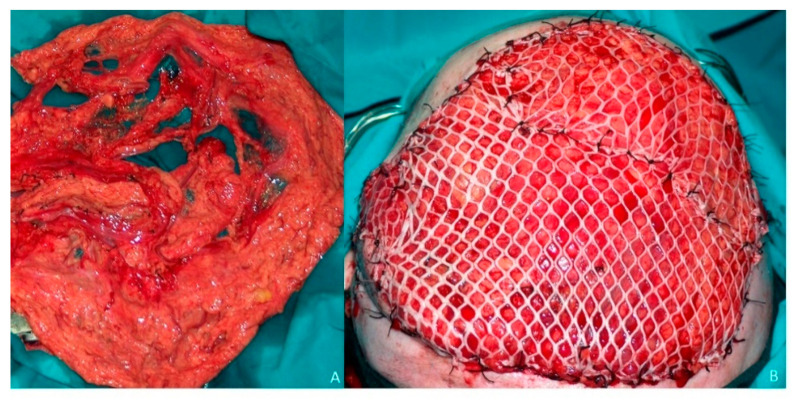
Omental flap. (**A**) Omental free flap harvested through laparoscopic approach pedicled to the right gastroepiploic vessels. (**B**) Scalp defect reconstruction with the omental free flap. Right gastroepiploic vessels anastomosed to the superficial temporal vessels. Omentum covered with a split-thickness skin graft.

**Figure 3:**
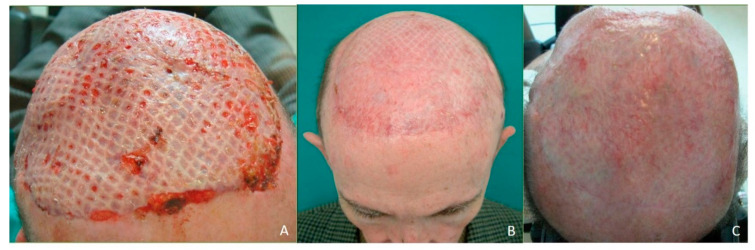
Omental flap. (**A**) Aesthetic result one-month post-operative. (**B**) Aesthetic result three-month post-operative. (**C**) Aesthetic result one year after reconstruction. No aesthetic distinction between the remaining scalp and the omental flap covered with skin graft. Good aesthetic result after extensive scalp reconstruction.

A 68-year-old patient diagnosed as squamous cell carcinoma of scalp. The lesion was resected and the patient underwent radiotherapy. Six months later the patient reported the appearance of several lesions in the scalp and recurrent carcinoma was diagnosed (Figure 1A). As a result of radiotherapy, the patient presented fibrosis and retraction of the scalp that prevented any type of reconstruction by means of local or regional flaps. The patient was operated on in a multidisciplinary surgery by means of a simultaneous two-team approach. On the one hand, extensive resection of the scalp was carried out together with a bilateral preauricular approach to identify and dissect the superficial temporal vessels (Figure 1B). Simultaneously, a laparoscopic approach was performed to harvest the omental flap. The vascular pedicle corresponding to the right gastroepiploic vessels was identified, the pedicle of the left gastroepiploic artery was ligated, leaving the omentum pedicled to the right gastroepiploic artery (Figure 2A). The omentum was then sutured to the remaining scalp and the anastomoses between the right gastroepiploic vessels and the superficial temporal vessels were performed. Finally, the omental flap was covered with a split-thickness skin graft (Figure 2B). From the aesthetic point of view, the patient was very satisfied with the result, as the marks from the mesh had over time disappeared from the skin and the tissue was uniform over the entire scalp (Figure 3A–C).

### 3.2. Latissimus Dorsi Flap

Ten patients were reconstructed with the myocutaneous LD flap. Seven patients were male (70%), and three patients were female (30%), with a mean age of 62.00 ± 3.54 years (range 33 to 71 years). Pathology included squamous cell carcinoma (n = 8), basal cell carcinoma (n = 1), and angiosarcoma (n = 1). The size of flaps ranged from 9 to 13 cm in width and 8 to 12 cm in length. The recipient vessels were the superficial temporal artery and vein (n = 5) and the facial vessels (n = 5). The survival rate of the flap was 100%. Four patients underwent postoperative radiotherapy (60 Gy). One patient was previously reconstructed with a rectus abdominis flap, but the flap failed due to venous thrombosis and the defect was reconstructed with a LD flap. One patient developed a seroma in the donor site and a wound dehiscence was reported in two patients. A two-team approach was only performed in two patients (20%) as a result of the patient’s position. The mean length of vascular pedicle was 12.34 ± 0.55 cm (range 9.9 to 15.1 cm). The mean operation time was 8.91 ± 0.32 h (range 7.8 to 10.4 h). The mean hospital stay was 17.90 ± 1.76 days (range 15 to 32 days) and the aesthetic result was reported as a very unsatisfactory in four patients (40%), unsatisfactory in four patients (40%), and a neutral result in two patients (20%) (Table 3 and Table 4).

#### Case Presentation

**Figure 4:**
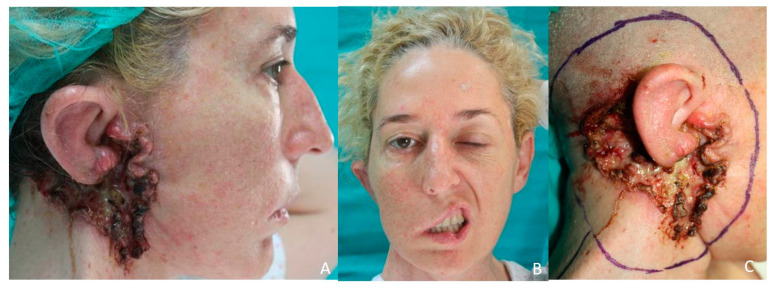
Latissimus Dorsi flap. (**A**) Preauricular basal cell carcinoma with auricular invasion and facial paralysis (**B**). (**C**) Design of the resection.

**Figure 5:**
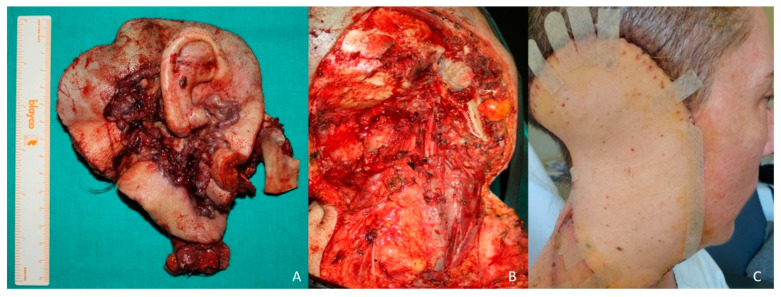
Latissimus dorsi flap. (**A**) Resection with clear margins including the ascending ramus of the mandible and the condyle. (**B**) Defect created after resection of the carcinoma including the ear, facial nerve, parotid gland, ascending ramus of the mandible and condyle. (**C**) Reconstruction with latissimus dorsi (LD) flap.

**Figure 6:**
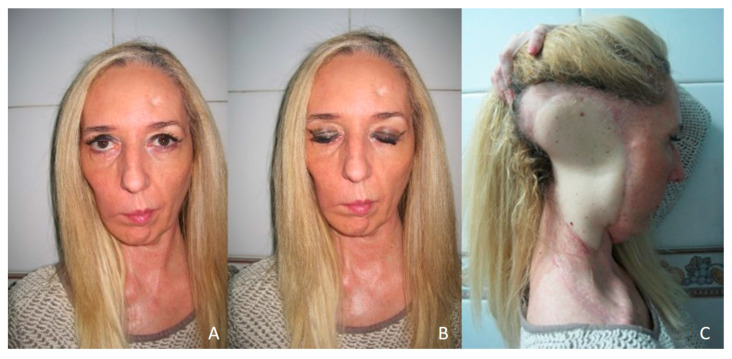
LD flap. (**A**,**B**) Aesthetic and functional result one year after surgery. (**C**) Poor aesthetic result one year after surgery. Significant difference in color and texture from the surrounding tissue.

A 33-year-old patient presented a 7 × 5 cm right preauricular basal cell carcinoma (Figure 4A–C). A CT scan was performed and revealed invasion of the facial nerve and invasion of the parotid gland and the ascending mandibular ramus. A resection of the tumor was performed (Figure 5A) with clear margins including the ear, parotid gland, scalp, facial nerve, mandibular ascending ramus, and condyle (Figure 5B). The facial vessels were identified at the cervical level for microsurgical anastomosis. The defect was reconstructed with a latissimus dorsi flap (Figure 5C). Five years after treatment, the patient does not present recurrence but reports a poor aesthetic result (Figure 6A–C).

### 3.3. Anterolateral Thigh Flap

Eleven patients were reconstructed with the ALT flap. Seven patients were male (63.6%), and four patients were female (36.4%), with a mean age of 65.82 ± 1.57 years (range 57 to 73 years). Pathology included squamous cell carcinoma (n = 10) and angiosarcoma (n = 1). The size of flaps ranged from 8 to 13 cm in width and 8 to 11 cm in length. The recipient vessels were the superficial temporal artery and vein (n = 6) and the facial vessels (n = 5). The survival rate of the flap was 100%. Six patients underwent radiotherapy (60 Gy), of which one was preoperative radiotherapy the rest postoperative radiotherapy. One patient developed a seroma in the donor site treated conservatively. A thrombosis of the venous anastomosis was identified 24 h after surgery in one patient and required an interpositional vein graft to perform an end-to-end anastomosis. In all patients, a two-team approach was performed. The mean length of vascular pedicle was 13.20 ± 0.26 cm (range 14.8 to 11.8 cm). The mean operation time was 7.53 ± 0.22 h (range 6.7 to 8.8 h). The mean hospital stay was 14.91 ± 0.60 days (range 13 to 19 days) and the aesthetic result was reported as a very unsatisfactory in 6 patients (54.4%), unsatisfactory in four patients (36.4%), and a neutral result in one patient (9.1%) (Table 3 and Table 4).

#### Case Presentation

**Figure 7:**
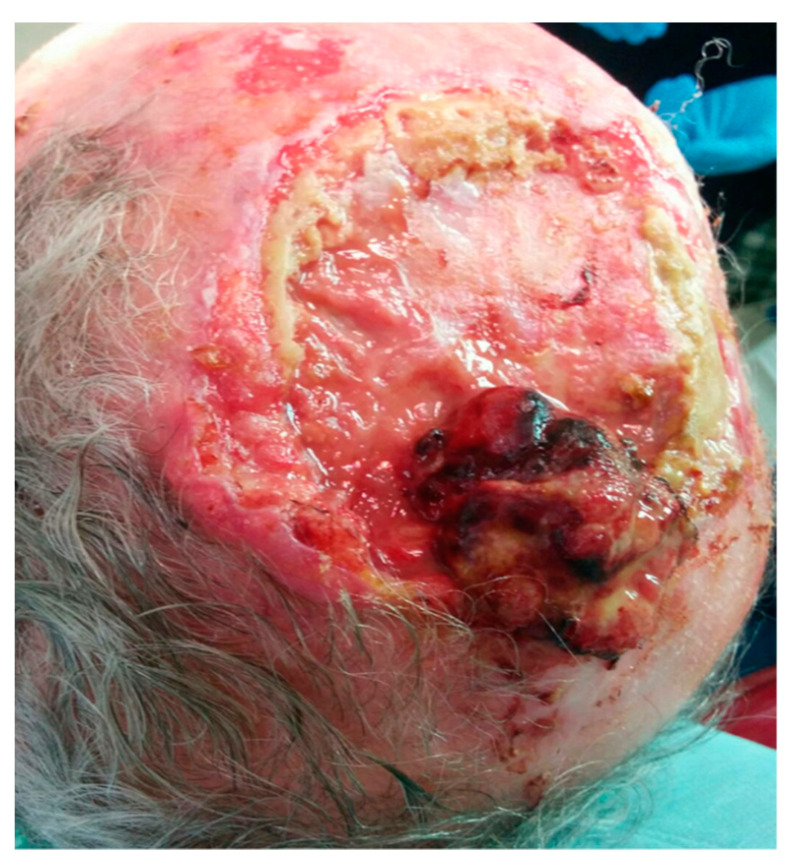
ALT flap. Squamous cell carcinoma of the parietal and temporal region.

A 64-year-old man was referred to our department for treatment of a squamous cell carcinoma of the scalp affecting the parietotemporal region. CT scan revealed a 13 × 9 cm tumor with no cranial invasion (Figure 7).

**Figure 8:**
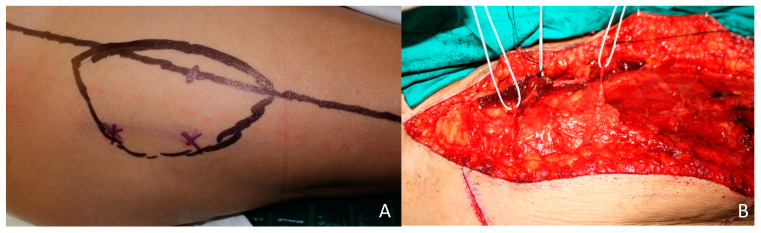
ALT flap. (**A**) Preoperative design of the ALT flap based in two perforators. (**B**) Musculocutaneous perforators identified during ALT flap dissection.

**Figure 9:**
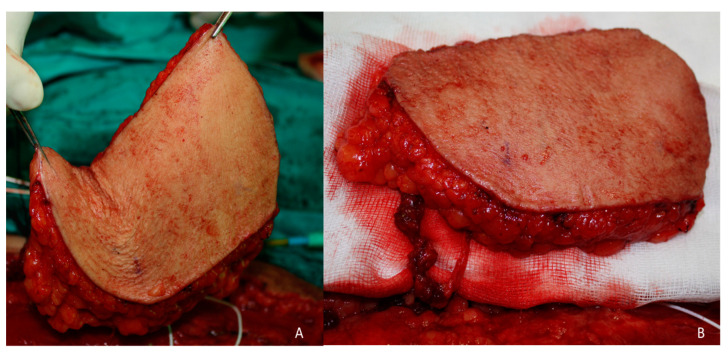
ALT flap. Anterolateral thigh flap based on two perforators prior to section of the vascular pedicle.

**Figure 10:**
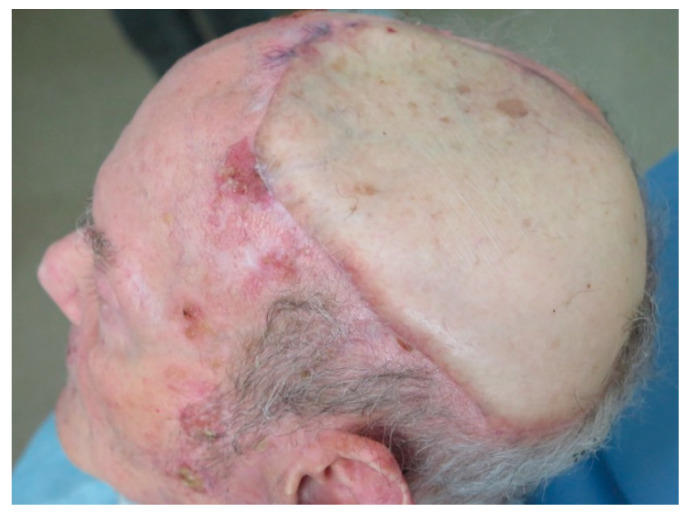
ALT flap. Follow-up four months after surgery with poor aesthetic result.

A wide resection with clear margins and dissection of the temporal vessels was performed. Simultaneously, an Anterolateral Thigh flap was harvested based in two musculocutaneous perforators. (Figure 8A). The vastus lateralis muscle was retracted laterally, the perforators were identified, and the pedicle was ligated distally and dissected proximally until a sufficient length of pedicle was obtained (Figure 8B and Figure 9A,B). The superficial temporal vessels were identified, and anastomosis were performed. No postoperative complications were observed, and the patient reported a very unsatisfactory aesthetic result during the follow-up (Figure 10).

The variables evaluated and compared between the three reconstructive techniques are shown in Table 3 and Table 4:

(A) Length of the vascular pedicle.

The OM flap was the flap with the shortest vascular pedicle length with a mean of 6.26 ± 0.16 cm, compared to the LD flap which was 12.34 ± 0.55 cm and the ALT flap with 13.20 ± 0.26 cm., statistically significant between OM flap and LD flap and ALT flap (*p* < 0.05). No significant differences were found between LD flap and ALT flap.

(B) Operation time.

The average time of surgery was 6.6 ± 0.14 h in patients reconstructed with OM, compared to the LD flap which was 8.91 ± 0.32 h and the ALT flap with 7.53 ± 0.22 h, statistically significant between the three groups (*p* < 0.05).

(C) Two-team approach.

A two-team approach was possible in all patients for OM flaps and ALT flaps. Due to the lateral position of the patients, the two-team approach could only be performed in two patients reconstructed with LD flap (*p* < 0.001).

(D) Aesthetic result.

The aesthetic result was evaluated, and the results were classified with scores 1 (“very unsatisfactory”), 2 (“unsatisfactory”), 3 (“neutral”), 4 (“satisfactory”), and 5 (“very satisfactory”). There was a significant difference in the aesthetic results reported. In patients reconstructed with the OM flap, a very satisfactory or satisfactory result was reported in seven patients (77.8%). In patients reconstructed with LD flap, eight patients reported a very unsatisfactory or unsatisfactory result (80%), and no patient reported a satisfactory aesthetic result. In a similar way, in patients reconstructed with ALT flap, 10 patients reported a very unsatisfactory or unsatisfactory result (90.9%) (*p* = 0.002). Attempts have been made to perform different multinomial regression models (dependent variable aesthetic results with five groups: very unsatisfactory, unsatisfactory, neutral, satisfactory, and very satisfactory”) as well as binary regression models (satisfactory aesthetic results: not/yes); however, it has not been possible due to limited sample size.

(E) Complications.

Two patients reconstructed with the OM flap developed partial skin graft failure solved with conservative treatment (22.2%). In patients reconstructed with LD flap, one patient developed a seroma and two patients developed wound dehiscence solved with conservative treatment. One patient required revision of the microvascular anastomosis (40%). In patients reconstructed with ALT flap, a seroma was developed by one patient and a venous thrombosis was encountered in one patient that required revision of the microvascular anastomosis (18.2%). The statistical analysis was not significant (*p* = 0.235).

(F) Length of hospital stay.

The mean hospital stay after surgery was 15.78 ± 0.93 days for patients reconstructed with the OM flap, 17.90 ± 1.76 days for LD flap, and 14.91 ± 0.60 days for ALT flap. These differences were not statistically significant (*p* > 0.05).

## 4. Discussion

Reconstruction of complex scalp defects after oncologic resection can present difficulties regarding functional and aesthetic outcomes. The reconstructive outline of scalp defects includes primary closure, split-thickness skin grafts, local and regional flaps, and tissue expanders [8,9,10,11]. Ideally, a like-to-like reconstruction is desirable. Nevertheless, microsurgical reconstruction is indicated in patients with large full-thickness defects of the scalp, cranial bone defects, and defects that involve dead spaces or in which previous reconstructive attempts with local or regional flaps were unsuccessful [1] and a salvage procedure is required. Both size and depth of defect have been recognized as critical factors dictating the need for free flap reconstruction [8,9]. The objectives of scalp reconstruction are: (1) to provide enough soft tissue to cover cranial bone exposure; (2) to provide soft tissue for aesthetic reconstruction to achieve an optimal result with a similar color and texture to the previous condition.

Free flaps for scalp reconstruction need to have several characteristics [4]: (1) sufficient tissue for extensive defects; (2) low donor site morbidity; (3) long vascular pedicle; (4) reliable and constant anatomy; (5) versatility in shape; (6) possibility of a two-team approach; and (7) good aesthetic results in terms of color and texture. Although the ideal flap for scalp reconstruction has yet to be described, omental flap, latissimus dorsi flap, and anterolateral thigh flap fulfill most of these characteristics and are widely used for complex scalp reconstruction. The aim of this retrospective study was to evaluate and compare the outcomes of scalp reconstruction in oncologic patients reconstructed with LD, ALT, and OM free flaps.

In terms of amount of tissue and versatility of shape, the three flaps widely comply with these requirements. The OM flap is a very pliable flap, which can be molded and folded to achieve adequate volume and can be adapted to reconstruct large and complex defects. Our study reported minor complications with no significance differences when using these flaps. Although severe complications have been described with the OM flap [12], the development of laparoscopic techniques have minimized these complications and it can be considered a safe and reliable procedure. When analyzing the pedicle length, LD and ALT flaps have a longer pedicle length than the OM flap (average 12.3 cm and 13.2 cm vs. 6.2 cm). This issue led us to perform the anastomosis to the superficial temporal vessels or to the facial vessels at a distal level in the facial area when using the OM flap. Therefore, it is difficult to achieve a suitable anastomosis at the cervical level when OM flap is used for reconstruction. On the other hand, its pliability allows the vascular pedicle to be positioned in the preauricular, facial, and upper cervical areas for microvascular anastomosis without difficulty. LD flap and ALT flap provide sufficient length of pedicle to perform the anastomosis at any cervical level.

Another important issue is the possibility of working in a two-team approach. One of the main advantages of a two-team approach is the possibility of being able to plan the oncologic resection and design the reconstructive flap according to the defect. All patients reconstructed with OM and ALT flaps were operated on a two-team approach. When using the LD flap, only two patients (20%) could be operated on simultaneously due to the patient’s change of position to lateral decubitus and this resulted in longer operation time. Analyzing this aspect, the statistical analysis showed significant differences in the surgical times between the three flaps. No significant differences were encountered in terms of hospital stay between techniques.

As for the aesthetic result, there were substantial differences between the different techniques. Patients referred a very unsatisfactory or unsatisfactory result when the reconstruction was performed with ALT flap or LD flap. Patients reported severe discrepancy in color and texture between the flap and the remnant surrounding tissue. Seven patients reported a very satisfactory or satisfactory aesthetic result, and one patient referred a neutral aesthetic result when using the OM flap as the reconstructive technique. This was due to several reasons. First, the resections performed were wide resections that included most of the scalp and the result was smooth, consistent, and homogeneous. Second, the dermo-epidermal graft was fully fitted and several months after surgery the aesthetic result was nearly identical with respect to the remnant tissue. Third, the atrophy occurred in the OM flap allowed an alignment with the surrounding tissue in terms of tissue thickness. Different authors harvest the LD flap as a muscular flap covered by a split thickness skin graft (STSG) reporting good aesthetic results [13,14,15,16] although the patients shown in the literature show a moderate discrepancy in color and texture from the remnant tissue. In our Department, we harvest the LD flap as a musculocutaneous flap for better postoperative monitoring of the flap. It is evident that the aesthetic result when using the ALT flap is poor. Besides, future studies comparing the aesthetic result between the OM flap and the LD flap covered by a STSG should be performed to address the better aesthetic result for scalp reconstruction. In our study, the investigators attempted to perform different multinomial regression models (dependent variable aesthetic results with five groups: very unsatisfactory, unsatisfactory, neutral, satisfactory, and very satisfactory) as well as binary regression models (satisfactory aesthetic results: not/yes); however, it has not been possible due to limited sample size.

To date, no report has addressed the comparison of the OM flap, the LD flap, and the ALT for extensive complex scalp reconstruction. Steiner reports [3] a retrospective study comparing the results between skin grafts, local and free flaps. They report radial, rectus abdominis, LD flaps, and one ALT flap but no OM flap is reported. Lutz and Wei [8] report a study of 30 patients reconstructed with either radial flap or LD flap. Chen [17] reports seven patients with large scalp defects reconstructed with ALT flap. Horn et al. [4] compare the ALT flap and the LD flap in soft tissue reconstruction of extensive defects in the head and neck. It compares the pedicle length, the flap size, the donor site morbidity, and the two-team-approach, but they do not compare the aesthetic result of scalp reconstruction, neither use the OM flap. In our opinion, fasciocutaneous flaps provide limited tissue for scalp reconstruction and the aesthetic result is poor, similar to that provided by the LD flap and the ALT flap [18,19,20,21]. The OM flap has several advantages for scalp reconstruction: (a) it provides a large amount of soft tissue to cover wide defects; (b) it is easy to shape in irregular edges over the bony surfaces; (c) high vascularity; (d) possibility of a two-team approach; (e) minor complications as a result of the minimally invasive approach; (f) its high pliability and adaptability allows to extend the vascular pedicle to the upper cervical region; (g) good aesthetic result when covered with skin graft. Nevertheless, it has two disadvantages: (1) it has a short vascular pedicle that can be compensated by the flexibility of the flap; (2) prior abdominal surgery may be a contraindication due to the risk of injury to the vascular pedicle. Its postoperative atrophy, although not ideal for the reconstruction of cranial defects, allows a good and homogeneous aesthetic result for scalp reconstruction. For all these reasons, we believe that the OM flap should be considered as a first line treatment flap as well as the LD flap and the ALT flap for reconstructions of extensive scalp defects in which there is no cranial bone defect. Although in this study patients reconstructed with OM flap did not receive postoperative radiotherapy, the authors consider that radiotherapy is not a contraindication for the use of the OM flap and the effect of radiotherapy on postoperative atrophy and its aesthetic outcome should be analyzed in future studies.

In patients with full-thickness defects in which cranial bone is resected and functional result is mandatory, titanium or PEEK patient specific implant should be considered simultaneously with LD flap and ALT flap to provide a large quantity of tissue that makes it possible to seal dead spaces, isolate the central nervous system, prevent chronic infections, and reconstruct associated soft tissues defects [19,20]. Both provide excellent soft tissue volume and a long vascular pedicle. Both flaps are easy to harvest with low donor site morbidity. Although the aesthetic result is poor in both flaps, the ALT flap has the advantage of being able to work in two teams simultaneously, which leads to a shorter operation time.

Therefore, which flap do we use to reconstruct extensive scalp defects to achieve a good clinical and surgical result for our patients? Our decisional workflow depends on the characteristics of the patients and the experience of the surgical team. In patients with previous abdominal surgery, the OM flap would be contraindicated, as well as the LD flap in previous axillary surgery. Considering that complications and hospital stay time are similar among the three flaps, we must take into account the availability and location of recipient vessels in order to select a flap with sufficient length of vascular pedicle. If the caliber of the temporal vessels is not good or we foresee that the anastomoses will be difficult due to the distance to the recipient vessels, the OM flap should be avoided because of its shorter vascular pedicle length. We must evaluate the possibility of working in two teams simultaneously and shorten the surgical time, in these cases, we must consider using the ALT flap or the OM flap. Moreover, from the esthetic point of view, if this aspect is a priority, we should consider reconstructing the defect with an OM flap or an LD flap covered with an STSG, avoiding the use of the ALT flap.

The main limitation of the present is the limited sample size. Further multicenter studies with larger sample sizes that allow performing multivariate analysis are needed to confirm our findings, as well as to evaluate impact of different variables on aesthetic results according to scalp reconstruction techniques and the long-term stability of the soft tissues. Due to the retrospective nature of the study, there is a selection bias since not all patients had the option of all three flaps. In some patients, flap options were limited because of contraindications for one or more of the techniques analyzed.

## 5. Conclusions

Omental flap, latissimus dorsi flap, and anterolateral thigh flap fulfill most of the characteristics for complex scalp reconstruction. The decision on which flap to use should be based on clinical aspects of the patients taking into account that the three flaps show similar rates of complications and length of hospital stay. If a long vascular pedicle length is required, LD flap or ALT flap should be considered. In patients in which OM flap is contraindicated, latissimus dorsi and ALT flaps are indicated due to the large volume they provide and, as a result of the possibility of a two-team approach and shorter operation time, the ALT flap seems to be superior to the LD flap. If aesthetic outcome is a priority, the OM flap should be considered for reconstruction of extensive scalp defects.

## Figures and Tables

**Table 1:** Descriptive continuous variables in total study population. Results represented as mean ± SEM.

Variables	Total Population (N = 38)
Age (years)	63.63 ± 1.38
Length of pedicle (cm)	10.83 ± 0.60
Operation time (hours)	7.71 ± 0.22
Hospital stay (days)	16.17 ± 0.70

**Table 2:** Descriptive categorical variables in total study population.

Variables	Categories	Total Population (N/%)
Gender	Male	20/66.7
	Female	10/33.3
Recipient vessels	Facial	11/36.7
	Superficial-temporal	19/63.3
Complications	No	21/70.0
	Yes	9/30.0
Radiotherapy	No	18/60.0
	Yes	12/40.0
2-team-approaching	No	8/26.7
	Yes	22/73.3
Aesthetic result	Very insatisfactory	10/33.3
	Insatisfactory	8/26.7
	Neutral	5/16.7
	Satisfactory	6/20.0
	Very satisfactory	1/3.3
Techniques	Omental flap	9/30.0
	Latissimus dorsi flap	10/33.3
	Antherolateral thigh flap	11/36.7

**Table 3:** Results represented as mean ± SEM. * *p* < 0.05 respect to omental flap group. ^#^ *p* < 0.05 respect to latissimus dorsi flap group.

Variables	Omental Flap (N = 9)	Latissimus Dorsi Flap (N = 10)	Antherolateral Thigh Flap (N = 11)
Age (years)	62.78 ± 1.59	62.00 ± 3.54	65.82 ± 1.57
Length of pedicle (cm)	6.26 ± 0.16	12.34 ± 0.55 *	13.20 ± 0.26 *
Operation time (hours)	6.60 ± 0.14	8.91 ± 0.32 *	7.53 ± 0.22 *^#^
Hospital stay (days)	15.78 ± 0.93	17.90 ± 1.76	14.91 ± 0.60

**Table 4:** Comparison of complications, radiotherapy, two-team approach, and the aesthetic result.

Variables	Categories	Omental Flap (N/%)	Latissimus Dorsi Flap (N/%)	Antherolateral Thigh Flap (N/%)	*p* Value
Complications	No	7/77.8	6/60.0	9/81.8	0.235
	Yes	2/22.2	4/40.0	2/18.2	
Radiotherapy	No	7/77.8	6/60.0	5/45.5	0.340
	Yes	2/22.2	4/40.0	6/54.5	
Two-team-approach	No	0/0	8/80.0	0/0	<0.001
	Yes	9/100	2/20.0	11/100	
Aesthetic result	Very insatisfactory	0/0.0	4/40.0	6/54.4	0.002
	Insatisfactory	0/0.0	4/40.0	4/36.4	
	Neutral	2/22.2	2/20.0	1/9.1	
	Satisfactory	6/66.7	0/0.0	0/0	
	Very satisfactory	1/11.1	0/0.0	0/0	

## Data Availability

The data presented in this study are available on request from the corresponding author. The data are not publicly available due to data protection regulations.

## Supplementary Materials

The following are available online at https://www.mdpi.com/article/10.3390/jcm10173863/s1, Table S1: Omental flap, Table S2: Latissimus dorsi flap, Table S3: Anterolateral thigh flap.
