# Supplementary material for: Clinical and Surgical Outcomes in Extensive Scalp Reconstruction after Oncologic Resection: A Comparison of Anterolateral Thigh, Latissimus Dorsi and Omental Free Flaps
Source: J Clin Med. 2021 Aug 27;10(17):3863. doi: 10.3390/jcm10173863 (PMC8432082; doi:10.3390/jcm10173863)
Supplement: Supplementary file 1 [file jcm-10-03863-s001.zip › jcm-1344961-supplementary.pdf]

TABLE S1. Omental Flap.

Age/Gender	Diagnose	Location defect	Flap size (cm)	Recipient vessels	Complications	Radiotherapy	2-team approach	Length of pedicle (cm)	Operation time (hours)	Hospital stay (days)	Aesthetic result
64/Male	Squamous cell carcinoma	Parietal-occipital	19x16	Superficial temporal	None	Yes (60 Gy)	Yes	6.3	6.8	13	4
60/Female	Squamous cell carcinoma	Frontal-parietal-temporal	18x19	Facial	Partial skin graft failure	No	Yes	5.8	7.5	21	4
58/Male	Basal cell carcinoma	Frontal-temporal-parietal	16x10	Superficial temporal	None	No	Yes	6.1	6.5	14	5
62/Female	Adenocarcinoma	Frontal-parietal	14x15	Superficial temporal	None	No	Yes	5.9	6.2	13	3
66/Male	Melanoma	Parietal-temporal	12x13	Superficial temporal	Partial skin graft failure	No	Yes	6.2	6.3	19	4

55/Male	Squamous cell carcinoma	Frontal-temporal-parietal	17x13	Superficial temporal	None	No	Yes	7.3	6.1	14	4
66/Female	Squamous cell carcinoma	Frontal-temporal-parietal	21x19	Superficial temporal	None	No	Yes	5.8	6.7	17	4
71/Male	Squamous cell carcinoma	Temporal-parietal	16x14	Superficial temporal	None	No	Yes	6.2	6.4	15	3
63/Male	Squamous cell carcinoma	Frontal-temporal-parietal	19x17	Superficial temporal	None	Yes (60 Gy)	Yes	6.7	6.9	16	4

TABLE S2. Latissimus Dorsi Flap.

Age/Gender	Diagnose	Location defect	Flap size (cm)	Recipient vessels	Complications	Radiotherapy	2-team approach	Length of pedicle (cm)	Operation time (hours)	Hospital stay (days)	Aesthetic result
64/Female	Squamous cell carcinoma	Fronto-parietal	9x10	Superficial temporal	Seroma	No	Yes	9.9	7.9	16	1
57/Male	Squamous cell carcinoma	Parietal-occipital	10x11	Superficial temporal	None	Yes (60 Gy)	No	11.3	8.7	15	2
71/Male	Squamous cell carcinoma	Temporal	8x10	Facial	Wound dehiscence	No	Yes	10.2	7.8	21	2
66/Male	Squamous cell carcinoma	Parietal-occipital	11x8	Superficial temporal	None	Yes (60 Gy)	No	12.8	8.5	12	3
68/Male	Squamous cell carcinoma	Temporal-parietal	12x8	Facial	Wound dehiscence	No	No	13.1	8.7	16	2

33/Female	Basal cell carcinoma	Temporal	9x8	Facial	Failure of prior rectus abdominis flap	No	No	11.5	10.4	32	1
63/Male	Angiosarcoma	Fronto-parietal	14x12	Superficial temporal	Revision microvascular anastomosis	No	No	15.1	10.3	19	1
70/Male	Squamous cell carcinoma	Fronto-temporal	13x12	Facial	None	Yes (60 Gy)	No	14.2	8.1	16	2
69/Male	Squamous cell carcinoma	Temporal-parietal	14x11	Facial	None	Yes (60 Gy)	No	13.9	8.5	14	3
59/Female	Squamous cell carcinoma	Fronto-parietal	16x13	Superficial temporal	None	No	No	11.4	10.2	18	1

TABLE S3. Anterolateral Thigh Flap.

Age/Gender	Diagnose	Location defect	Flap size (cm)	Recipient vessels	Complications	Radiotherapy	2-team approach	Length of pedicle (cm)	Operation time (hours)	Hospital stay (days)	Aesthetic result
70/Male	Squamous cell carcinoma	Frontal-Parietal	11x9	Superficial temporal	None	Yes (60 Gy)	Yes	13.8	7.2	13	2
58/Male	Squamous cell carcinoma	Temporal	7x5	Facial	None	No	Yes	12.9	6.7	14	1
73/Female	Squamous cell carcinoma	Frontal-parietal	12x10	Superficial temporal	Seroma	Yes (60 Gy)	Yes	12.3	7.8	16	1
66/Male	Squamous cell carcinoma	Temporal	10x9	Facial	None	No	Yes	13.3	6.9	14	1
57/Female	Angiosarcoma	Frontal-temporal	9x8	Facial	None	Yes (60 Gy)	Yes	12.8	8.8	18	1

67/Male	Squamous cell carcinoma	Frontal-temporal	13x9	Facial	Venous thrombosis	Yes (60 Gy)	Yes	14.8	6.7	19	1
65/Male	Squamous cell carcinoma	Parietal	8x8	Superficial temporal	None	No	Yes	14.3	6.8	14	2
69/Female	Squamous cell carcinoma	Parietal	11x7	Superficial temporal	None	Yes (60 Gy)	Yes	12.6	7.7	13	3
72/Male	Squamous cell carcinoma	Parietal-temporal	9x8	Facial	None	No	Yes	13.4	8.3	14	2
63/Female	Squamous cell carcinoma	Parietal	8x9	Superficial temporal	None	No	Yes	11.8	8.1	15	2
64/Male	Squamous cell carcinoma	Parietal-temporal	11x10	Superficial temporal	None	Yes (60 Gy)	Yes	13.2	7.8	14	1
